# Coproduction of an occupation-based complex intervention for living well with anxiety and Parkinson’s (OBtAIN-PD) using online logic modelling in the UK

**DOI:** 10.1136/bmjopen-2025-107930

**Published:** 2026-02-22

**Authors:** Chris Lovegrove, Katrina Bannigan, Jonathan Marsden, Ingrid Sturkenboom

**Affiliations:** 1 NIHR Newcastle PSRC, Newcastle University Faculty of Medical Sciences, Newcastle upon Tyne, UK; 2 Occupational Therapy Department, Newcastle Upon Tyne Hospitals NHS Trust, Newcastle upon Tyne, UK; 3 School of Health Professions, University of Plymouth, Plymouth, UK; 4 School of Health and Wellbeing, University of Glasgow, Glasgow, UK; 5 Donders Institute for Brain, Cognition and Behavior; Department of Rehabilitation, Radboud University Medical Center, Nijmegen, Netherlands

**Keywords:** Parkinson-s disease, Health, MENTAL HEALTH, Patient Participation, Anxiety disorders, QUALITATIVE RESEARCH

## Abstract

**Abstract:**

**Background:**

Anxiety is a common non-motor symptom of Parkinson’s disease (PD). There is no specific pharmacological intervention for people with PD who experience anxiety. Current non-pharmacological treatments have mixed or inconclusive results and there does not appear to be a non-pharmacological intervention for people with PD disease and anxiety that focuses on activity and participation.

**Objective:**

To co-produce an occupation-focused complex intervention to help people with PD live well with anxiety that community-based occupational therapists can deliver.

**Design:**

Six-stage complex intervention development was conducted using online logic modelling and a participatory approach to organise the new intervention’s key inputs, processes and outcomes important to people with PD living with anxiety.

**Setting:**

Data were collected via online logic modelling sessions involving people with Parkinson’s, care partners and occupational therapists across the UK from April 2022 to June 2022.

**Participants:**

34 participants were recruited (people with PD n=14, care partners n=9, occupational therapists n=11) for the online logic modelling sessions.

**Results:**

Resources to support the new intervention (‘inputs’) include adequate resourcing, education for professionals and people with PD, flexibility of delivery methods and goal setting. The intervention’s actions to produce outcomes (‘processes’) should include 1:1 support, lifestyle management, providing meaningful information, collaborative goal setting, therapeutic use of everyday activities, and involvement of friends and families. The intended results (‘outcomes’) should include a reduction in anxiety symptoms, people with PD enjoying more meaningful activities, increased understanding of anxiety and PD, improvement in clinical outcomes and improvement of service-level outcomes. These key aspects were incorporated into an intervention manual, educational material and training video.

**Conclusions:**

We have systematically coproduced a new occupation-focused complex intervention to help people with PD to live well with anxiety. This provides the basis for the next project in which this intervention will be tested for feasibility.

**Trial registration number:**

ISRCTN62762494.

**STRENGTHS AND LIMITATIONS OF THIS STUDY**

- The coproduction approach benefits from a structured process where participants contribute directly through logic modelling.
- Online sessions allowed for a convenient participation method that promoted engagement.
- We tailored our engagement in the sessions to meet the needs of individual groups for this research project, whether through group or individual sessions scheduled at convenient times for participants.
- The under-recruitment of care partners reflects the systemic barriers they face in research participation, including time constraints arising from their caregiving duties.
- Our sample across all groups primarily consisted of white English individuals from Christian or non-religious backgrounds, with other ethnic and religious denominations represented in smaller numbers, thus limiting external validity.

## Introduction

 Parkinson’s disease (PD) is the second most common neurodegenerative condition worldwide and is diagnosed based on the presence of motor symptoms like bradykinesia, along with either resting tremor or rigidity, and the exclusion of other conditions.^1^ Globally, the prevalence of PD has doubled in the past 25 years and now affects more than 10 million.^2 3^ Recent estimates suggest that PD resulted in 5.8 million disability-adjusted life-years in 2019.^4^ Most people with PD (98.6%) live with non-motor symptoms in combination with motor symptoms.^5 6^ Between 43% and 56% of people with PD experience anxiety.^5 6^ Reviews have found a higher prevalence of anxiety in people with PD compared with the general population.^7^ People with PD and anxiety have a greater risk of falling, live with a greater health burden and have reduced quality of life, independence and social roles.^8 9^ Heightened anxiety in people with PD leads to increased fluctuations in motor symptom presentation.^6 10 11^

Anxiety is a continual internal feeling of worry and fear that intrudes on everyday life.^12^ People with PD experience more anxiety than those with other neurodegenerative conditions and fear negative public perceptions.^7^ People with PD may be more at risk of anxiety than those with other long-term conditions due to the dopamine deficiency that is characteristic of PD.^13^ Dopamine modulates the inhibitory mechanisms that the medial prefrontal cortex exerts on the anxiogenic output of the amygdala, a brain structure involved in anxiety.^14 15^ Dopamine deficiency leads to neuronal hyperexcitability and amplified responses to perceived adverse threats.^13 16 17^ Dopamine-replacement medication is a primary intervention in PD, but people with PD in the later stages can experience a notable increase in symptoms when the medication effects wear off between doses.^18^ Psychological stressors associated with long-term conditions experienced by people with PD may increase anxiety further.^19^ This contributes to the maintenance of the hyperexcited neuronal anxiety circuit.^16 17^ This suggests that people with PD experience anxiety differently from other populations because of the neurobiology specific to PD combined with the psychological stressors of living with an incurable long-term condition, and thus warrants an intervention that targets the needs of people living with PD specifically. The evidence for the use of psychological therapies, such as cognitive behavioural therapy, is mixed, and there is

currently no evidence-based occupational therapy intervention to help people with PD live well with anxiety.^20^,^23^ Occupational therapists are commonly involved in the treatment of people with PD, addressing activities and participation (ie, meaningful occupation).^24^,^26^ An occupation-focused intervention is one where the therapeutic approach is centred on enabling a person to engage in meaningful occupations—the everyday activities that bring purpose, identity and structure to life.^27^ Since anxiety impacts the experience or performance of occupation, occupational therapists must adjust their interventions to the specific needs of people with PD and anxiety.^28^ Evidence suggests promise for non-pharmacological approaches such as lifestyle management delivered by occupational therapists in populations not living with PD,^29^,^33^ highlighting a valuable direction for clinicians and researchers when developing complex interventions for people with PD experiencing anxiety, especially in the absence of a specific pharmacological treatment.^15 34^

The presence of anxiety in PD has an adverse effect on PD motor symptoms. Anxiety has been shown to increase the freezing of gait (FOG) episodes in ‘freezers’ and higher motor symptom severity when compared with ‘non-freezers’, implying that anxiety is an important mechanism underpinning FOG.^11^ This means that if people with PD are anxious, this will affect their functional mobility in daily life and consequently reduce their quality of life.^6 11 35^ Cognitive impairment associated with anxiety impacts quality of life.^35 36^ Other potential sources of stress that contribute to anxiety for all people, not just people with PD, include (but are not limited to) health, family, finances and work, all of which can change over the life course.^37^ Research suggests that anxiety is not an isolated experience but rather a complex interplay of multidimensional interactions with the world, often requiring tailored interventions.^38 39^

The charity Parkinson’s UK identifies anxiety as a research priority.^40^ Previous studies have contributed to understanding the experience of anxiety for people with PD and what sorts of concepts may be included in a new complex intervention.^3841^,^43^ People with PD have stated that they want an intervention aimed at living well with the complex experience of anxiety with PD, focused on ‘doing’ (ie, participation in meaningful occupation) as the means and goal of intervention rather than only talking about their thoughts and feelings.^38 41^

Occupational therapy focuses on enabling people with PD to participate in their chosen meaningful roles and activities.^44^ Occupational therapy can be delivered in a variety of settings dependent on the person with PD’s need, including acute, subacute and community-based contexts. Access to occupational therapy is recommended in clinical guidelines to support symptom management, promote independence and reduce hospital admissions.^45^ Research suggests that occupational therapy focused on the person’s prioritised meaningful goals in activities in daily living and participation is an effective intervention for people with PD.^46 47^ However, in the UK and Europe, it is estimated that just over half of people with PD report having consulted an occupational therapist to address their concerns about living with Parkinson’s.^48^ There is no occupational therapy intervention specific to people with PD with anxiety-related participation problems.

This study aimed to systematically coproduce an occupation-focused intervention centred on ‘doing’ that occupational therapists can deliver to help people with PD live well with anxiety.

## Methods

### Participants

The coproduction was with all potential end users: people with PD, care partners (the preferred term used by a person providing informal care or support for a person with PD), and occupational therapists. The recruitment target was n=30, with the sample split equally among people with PD, care partners and occupational therapists (10 participants per group). 12 weeks were allocated for recruitment.

People with PD and care partners were recruited purposively using a maximum variation strategy to maximise sample diversity across the stages of disease progression: diagnosis, maintenance, complex and palliative stages.^49 50^ Occupational therapists were recruited via clinical interest groups. People with PD and care partners were recruited via Parkinson’s UK’s ‘Research Support Network’.^51^ Snowball sampling helped access participants from under-represented groups who use health or social care services but are seldom heard by these services and decision-makers.^52^ We collected demographic data based on the UK Census to verify participant diversity.^53^

Occupational therapists were primarily recruited via the Royal College of Occupational Therapists Specialist Sections for ‘Neurological Practice’ and ‘Mental Health’. In addition, snowball sampling was used to recruit occupational therapists who may not be part of these groups but may have relevant experience in either PD or anxiety-related occupational therapy interventions.^52^

### Patient and public involvement

A patient and public involvement (PPI) representative was a research team member involved in developing the overarching logic model. In collaboration with people with PD and care partner PPI representatives, the information pack provided to participants was developed. Occupational therapist PPI representatives contributed to creating the intervention manual.

### Design

We used the Medical Research Council’s guidance for developing and evaluating complex interventions as a framework.^34^ This study focused on the development stage and was conducted in the UK. We used the method of logic modelling and a participatory approach to organise the new intervention’s key inputs, processes and outcomes for intervention development.

A logic model is a hypothesised description of the chain of causes and effects that result in an outcome of interest. Logic modelling is a systematic and visual way to present the relationships between an intervention programme’s resources, activities, outputs and outcomes. It helps in planning, implementing and evaluating programmes by clearly illustrating how the programme is intended to work. By mapping these components, logic models help stakeholders understand the intervention’s structure and expected impact. In this research, the end outcome is enabling people with PD to live well with anxiety by increasing their participation in meaningful activities. Logic modelling can involve representative stakeholders in a structured process to support complex interventions’ design, planning, communication and evaluation.^54^ Engaging diverse stakeholder groups in logic modelling can help make research more relevant and generalisable to real-world settings by making the findings more applicable to broader audiences. There were six stages in developing the occupation-based complex intervention for living well with anxiety and Parkinson’s disease (OBtAIN-PD) using the logic model.^55^

#### Stage 1: installing a project team and formulating key objectives

The core project team consisted of the research team (CL, KB, JM and IS) and a PPI representative. The core project team consisted of an occupational therapist and PhD student (CL), an occupational therapy researcher (IS) with expertise in Parkinson’s and complex intervention development and evaluation, a professor who is an occupational therapist with mental health and complex intervention development expertise (KB), a professor who is a physiotherapist with expertise in Parkinson’s, neurological rehabilitation and neuroscience (JM), and a person living with Parkinson’s. Additional advice was received from a professor of mental health services research, who is a nurse by background with expertise in mental health and the development of complex interventions. The key objective was to coproduce a new occupation-focused intervention to help people with PD live well with anxiety, considering recent research and individual lived experience that community-based occupational therapists could deliver.

#### Stages 2 and 3: component consensus and clustering of clinical activities into a process flow

Interventions delivered by occupational therapists, the intervention features and group consensus identified in previous research were used as input for modelling.^3841^,^43^ Participants were given an electronic or printed information pack (depending on participant choice) to inform their thinking. The information pack contained summaries of the findings from previously completed studies by the research team, including the outcomes of a scoping review examining occupational therapy interventions for community-dwelling adults with anxiety.^38 42 43^ The information pack had been refined in collaboration with PPI representatives.

Stages 2 and 3 co-occurred within the same session to reduce the time burden on participants attending multiple sessions. A PhD student researcher (CL, who received training as part of a doctoral fellowship) facilitated the logic modelling process, starting with long-term outcomes and moving backwards towards inputs. Sessions lasted 57 min on average. Each participant attended one logic modelling session. During the sessions, participants were invited to cluster clinical activities based on the extent to which the activities fit together (stage 2). The participants’ ideas were mapped directly onto a process flow diagram to illustrate the interaction of the individual components and their intended output by participants (stage 3). Participants were allowed to complete sessions in person or via an online platform (Zoom).^56^ Sessions were recorded as it was likely that the planned outputs (logic models) produced by the groups would have variations requiring further analysis by the research team. Individual logic models were completed at each group session and then combined into an overarching model by the research team with PPI involvement.^34 55 57^

Participants who felt unable to participate in a group session were offered a one-to-one session (n=2). All sessions were completed at a time, date and setting convenient for the participants. The people with PD, care partners and occupational therapists met separately to avoid potential bias caused by power dynamics.^49^ There was no set order for the different participant sessions to be completed, as each session produced an individual logic model.

#### Stage 4: process organisation

The final logic model was reviewed by participants from all groups (if they chose to take part) to consolidate the sizeable model into a consolidated, overarching logic model. This process involved combining each model into a final version, considering individual assumptions and potential external factors. This consolidated logic model was checked with two National Health Service (NHS) community therapy service managers to evaluate the organisational impact of the intervention. This took the form of a PhD student researcher (CL) presenting the logic model to the managers, providing an opportunity for them to comment on its feasibility and implementation within an NHS setting. This stage was conducted remotely for convenience. This information was used in subsequent stages.

#### Stage 5: detailed description of key interventions

A detailed protocol for the OBtAIN-PD intervention was written based on the data collected from stages 2–4. Individual components—the activities, inputs and outcomes identified by participants—were reconsidered.^55^ The research team completed this with input from PPI representatives.

#### Stage 6: translation into a set of process and outcome indicators

Process and outcome indicators were developed to help verify that the intervention was delivered as planned and intended.^58 59^ The research team completed this with input from PPI and developed it into an intervention manual. A final review with the occupational therapist group was conducted to check the content and comprehension of the intervention manual and inform the development of a final version.

## Results

The final sample was n=34. People with PD and their care partners participated in stages 2 and 3. Occupational therapists participated in stages 2, 3 and 6. The recruitment target of 10 in each stakeholder group was exceeded for people with PD (n=14) and occupational therapists (n=11), and below the target for care partners (n=9). 16 participants (people with PD (n=6), care partners (n=5) and occupational therapists (n=5)) consolidated the raw logic model. The demographic data of the participants are shown in table 1. Education status was not collected at the request of PPI representatives, who felt it was irrelevant to the research question.

**Table 1:** Participants’ demographic characteristics

Characteristic		All participants (n=34)	People with Parkinson’s (n=14)	Care partners (n=9)	Occupational therapists (n=11)
Gender	Male (including transgender men)	11 (32%)	6 (43%)	4 (44%)	1 (9%)
	Female (including transgender women)	22 (65%)	7 (50%)	5 (56%)	10 (91%)
	Prefer not to say	1 (3%)	1 (7%)	0	0
Age	25–34 years*	4 (12%)	0	1 (11%)	3 (27%)
	35–44 years	13 (38%)	3 (21%)	5 (56%)	5 (45%)
	45–64 years	9 (26%)	5 (36%)	1 (11%)	3 (27%)
	65–74 years	6 (18%)	5 (36%)	1 (11%)	0
	75 years and above	2 (6%)	1 (7%)	1 (11%)	0
	Median years PD diagnosis duration (range)	N/A	5 (1–22)	N/A	N/A
Employment status	Working as an employee	19 (56%)	5 (36%)	4 (44%)	10 (91%)
	Self-employed or freelance	3 (9%)	2 (14%)	1 (11%)	0
	Retired	9 (26%)	6 (43%)	3 (21%)	0
	Long-term sick or disabled	2 (6%)	1 (7%)	1 (11%)	0
	On maternity or paternity leave	1 (3%)	0	0	1 (9%)
Annual income (£GBP)	£20 001 to £30 000	3 (9%)	0	1 (11%)	2 (18%)
	£30 001 to £40 000	4 (12%)	1 (7%)	1 (11%)	2 (18%)
	£40 001 to £50 000	7 (20%)	2 (14%)	1 (11%)	4 (36%)
	£50 001 to £60 000	1 (3%)	1 (7%)	0	0
	£70 001 to £80 000	2 (6%)	1 (7%)	0	1 (9%)
	Prefer not to say	17 (50%)	9 (64%)	6 (67%)	2 (18%)
Marital status	Single/never married	5 (15%)	2 (14%)	3 (33%)	0
	Married/domestic partnership	23 (67%)	10 (71%)	4 (44%)	9 (82%)
	Divorced	6 (18%)	2 (14%)	2 (22%)	2 (18%)
Sexual orientation	Straight or heterosexual	30 (88%)	12 (86%)	7 (78%)	11 (100%)
	Gay or lesbian	4 (12%)	2 (14%)	2 (22%)	0
Religion	No religion	17 (50%)	5 (36%)	5 (56%)	7 (64%)
	Christian	11 (32%)	5 (36%)	2 (22%)	4 (36%)
	Jewish	3 (9%)	2 (14%)	1 (11%)	0
	Muslim	2 (6%)	1 (7%)	1 (11%)	0
	Any other (not described)	1 (3%)	1 (7%)	0	0
Ethnicity	White English, Welsh, Scottish, Northern Irish, or British	27 (79%)	9 (64%)	7 (78%)	11 (100%)
	Asian or Asian British	2 (6%)	1 (7%)	1 (11%)	0
	Black, African, Caribbean or Black British-African	3 (9%)	2 (14%)	1 (11%)	0
	Other ethnic group-Arab	1 (3%)	1 (7%)	0	0
	Any other ethnic group	1 (3%)	1 (7%)	0	0

* No participants younger than this were involved in the study

### Inputs for the intervention

Participants identified the resources needed to support the intervention and generated 58 statements based on their ideas. These were consolidated into five inputs: ‘Resources’, that is, the need for proper funding and staffing (specifically occupational therapists and the associated support staff), physical resources such as assessment equipment, and time to deliver the intervention and associated administration; ‘Education for professionals’ about PD; ‘Education for people with Parkinson’s’ about anxiety and how it affects PD; ‘Flexible delivery’ of the intervention in a variety of settings, including in-person and remotely and by occupational therapists and ‘Goal setting’ by participants from each group. Considering the causal chain, these were considered essential by participants to produce the activities of the intervention and achieve the desired outcomes.

### Processes of the intervention

The processes included what needed to be done as part of the intervention (activities) and who and what should be targeted by the intervention (participation). Participants from each group generated 103 statements, again based on their ideas, identifying the processes and activities they felt should be included in the new intervention. Participants from each group consolidated these statements into six aspects: ‘1:1 support first’ meaning 1:1 support should be prioritised over group work; ‘Lifestyle changes’ towards a healthier lifestyle to live well with anxiety; ‘Providing meaningful information’ that is concise and up-to-date, ‘Collaborative goal setting’ rather than the therapist leading, ‘Use everyday activities’, that is, using the person’s meaningful activities therapeutically and ‘Involve friends and families’ (where needed). The occupational therapists and managers believed that e-learning would be a suitable training method; however, they preferred face-to-face delivery for topics that involved highly advanced concepts. The group did not specify how much training would be necessary.

### Outcomes of the interventions

Participants generated 153 statements about the expected short-term, medium-term and long-term outcomes of the new intervention. Participants from each group consolidated these statements into the person with PD ‘Enjoying more meaningful activities’; ‘Better understanding’ of one’s anxiety and how to manage it; ‘Reduce anxiety symptoms’ on validated measures; ‘Improvement of clinical outcomes’ using validated occupational therapy measures, such as the Canadian Occupational Performance Measure (COPM), to assess the effectiveness of the intervention and ‘Service level outcomes’ such as reductions in new or re-referrals into services.

**Figure 1:**
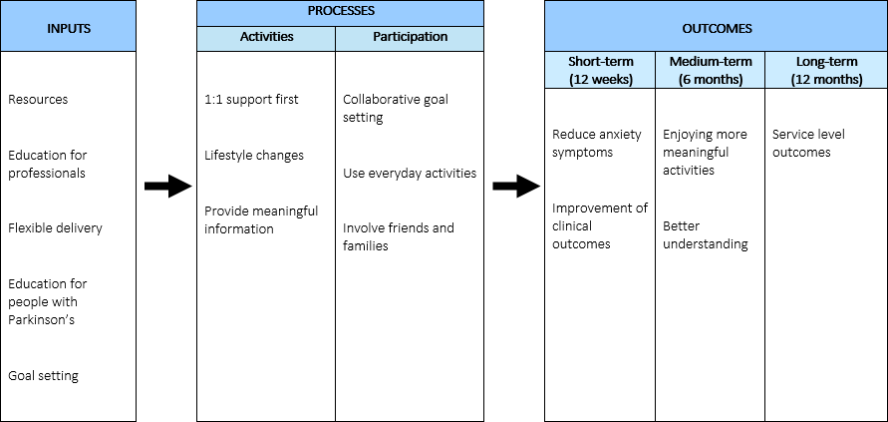
The consolidated logic model.

The consolidated logic model is presented in figure 1. Illustrative verbatim quotes are shown in table 2.

**Table 2:** Illustrative quotes from the consolidated logic model

Inputs	Resources	“Staffing availability as a resource. Physical resources; easy access to equipment, services, groups.” (OT)
	Education for professionals	“We need more people with more knowledge. Help in more areas. Increased knowledge amongst those people who have most contact with PWPs.” (person with PD)
	Education for people with Parkinson’s	“Education. Information that anxiety makes the physical symptoms of Parkinson's worse. Acknowledging it is okay to be anxious.” (person with PD)
	Flexible delivery	“There needs to be options for delivery. Some people don't have online access, some do.” (care partner)
	Goal setting	“Goal setting resources will be important.” (care partner)
Processes	1:1 support first	“1:1 basis for the sessions to be delivered.” (person with PD)
	Lifestyle changes	“Developing understanding between the links between lifestyle and anxiety. Understanding that you won't get everyone. You need to see the benefits/ feel-good factor of what you are doing. Developing a positive snowball effect. Using real-life examples to demonstrate benefits.” (person with PD)
	Providing meaningful information	“Working with the persons partner to help the PWP do things so they do not become inactive and building up the confidence of the carer.” (person with PD)
	Collaborative goal setting	“Goal setting- SMART goals used in o/p service- this works as a 'contract’ as well between the service and patient.” (OT)
	Use everyday activities	“The intervention has to focus on what is important to the person. Everyone is individual.” (care partner)
	Involve friends and families	“Working with the persons partner to help the PWP do things so they do not become inactive and building up the confidence of the carer.” (person with PD)
Outcomes (short, medium and long)	Reduce anxiety symptoms	“Really important to measure anxiety symptoms, important to show the person that it has reduced, and I have a reading to show that.” (OT)
	Enjoying more meaningful activities	“Participating more in activities, and hobbies, that they enjoy doing.” (care partner)
	Better understanding	“Have understanding of what anxiety is, had an opportunity to practise some coping strategies and then call on those a year down the line. The carers would be able to draw on these skills as well, and be able to draw on support when needed and not start at the beginning of the process.” (person with PD)
	Improvement of clinical outcomes	“COPM improvement—includes goals and breaks them down in a really specific way. Not a massive additional time constraint.” (OT)
	Service level outcomes	“No referral back to the service at the 6 month timepoint—may have actually referred on to another service (long term conditions counselling team).” (OT)

### Process flow

The consolidated logic model was presented to three NHS managers leading community rehabilitation teams. They felt the proposed model was feasible within current practice, and no further changes were suggested.

### Intervention manual

Following the review with NHS managers, the research team drafted an intervention manual, information sheets, and a collaborative goal-planning document, all with PPI input and based on the consolidated logic model. A review was conducted with occupational therapists from the sample (n=5) to assess the manual’s content and readability (online supplemental material 1). Based on feedback, a training video was developed to accompany the intervention manual.

## Discussion

This study aimed to systematically coproduce an intervention that occupational therapists can deliver to help people with PD live well with anxiety using a logic modelling approach.^55^

All participant groups stated that having access to adequate resources, such as time, staffing or physical materials, was necessary for the OBtAIN-PD. Resource limitations are associated with changes in care delivery and poorer clinical outcomes in some cases.^60^ Lack of resources is an identified barrier to intervention implementation in PD and other neurological conditions.^61 62^ The NHS is recognised as a resource-constrained environment, especially in community health services.^63^ The OBtAIN-PD must have a low-resource burden to be successfully implemented using only existing resources.^64 65^ This may include using existing outcome measures, minimising the impact on clinicians’ time, and providing flexibility on how the intervention can be delivered.^66^ For example, all participants highlighted providing adequate education to people with PD and occupational therapists, as necessary. Training and education can be time-consuming if not delivered appropriately, creating implementation barriers.^66^ Training for the OBtAIN-PD, for both people with PD and occupational therapists, must be concise and convenient; this could be addressed via e-learning.^67^

The participants in this study highlighted the importance of flexibility in how and when the OBtAIN-PD can be delivered. Previous research has shown that the ability to adapt the delivery mode of interventions and their timing can facilitate complex interventions, as community-based clinicians manage their own caseloads.^66^ Ensuring that the intervention can be delivered flexibly but with fidelity of the key components (by providing an intervention manual), supporting occupational therapists’ caseload demands, and the needs of people with PD will facilitate the successful implementation of OBtAIN-PD.

The occupational therapists did not express a preference for either group or individual intervention sessions. This is notable considering the advantages of group work in addressing service demands. However, people with PD and the care partners who participated in this study indicated that the OBtAIN-PD should primarily be delivered as a personalised individual intervention rather than through group sessions. Previous studies have reflected on the treatment preferences of people with PD.^41 68 69^ This suggests that the OBtAIN-PD should be delivered as a tailored individual intervention. It is common for interventions to adopt a multidisciplinary approach in managing long-term conditions, which can provide benefits such as efficient resource use and enhanced communication.^70^ On the other hand, this can require complex coordination and may result in role confusion.^70^ When coproducing the OBtAIN-PD, people with PD wanted an intervention that could be delivered flexibly in the community, and the occupational therapists felt that an intervention that could be delivered directly by themselves when required as part of their intervention toolkit would lead to better outcomes. Subsequently, OBtAIN-PD was designed as a specific occupational therapy intervention because the focus is on ‘doing’ in meaningful activities. It can be delivered as a single intervention or in combination with other interventions in the multidisciplinary team.

All participants agreed that lifestyle changes should be included in the OBtAIN-PD, as these changes could help people with PD manage anxiety and reduce symptoms. Preliminary research exists on lifestyle management approaches for people with PD, and more research is needed.^71 72^ Lifestyle management strategies aim to educate and motivate individuals to enhance their quality of life through healthier habits and daily behaviours.^33^ These approaches have been applied in various contexts to encourage engagement in meaningful activities.^33 73^ Similar approaches have been used in occupational therapy interventions, including for adults with generalised anxiety disorder.^29 33 74^ This approach aligns with our study participants’ opinion that the OBtAIN-PD should include providing meaningful information, collaborative goal setting, and the therapeutic use of everyday activity. These concepts are pillars of lifestyle management approaches.^33 73^ The provision of up-to-date, meaningful information, such as education about Parkinson’s and how lifestyle can influence anxiety, was particularly important to participants. Such information provision is important in empowering people to make informed choices about their lifestyle through knowledge acquisition and has been a supportive factor in the management of other conditions.^75 76^ Furthermore, lifestyle management approaches for long-term conditions often involve collaboration with care partners such as friends and families.^73 77^ The fact that lifestyle management approaches resonate closely with our participants’ opinions affirms the inclusion of these concepts in the OBtAIN-PD, which provides a new approach for occupational therapists to help people with PD live well with anxiety specifically.

Theoretically, this approach aligns with the ‘Do-Live-Well’ framework, an occupation-based health promotion framework designed to promote evidence-based links between participation in everyday activities and health and well-being.^78^ This model emphasises that ‘what you do every day matters’ and encourages reflection on how participation in daily activities (like cooking, working and socialising) can positively influence health and quality of life. Occupational therapists often use this model to assess and address a person’s needs through occupation-based interventions.^79 80^

Outcome measure selection is essential for comprehensively evaluating complex interventions.^34^ Establishing what outcomes an intervention aims to achieve allows the selection of appropriate outcome measurement tools.^81^ Participants from all groups stated that anxiety reduction is an essential outcome of the intervention and that they felt this was part of ‘living well’ with anxiety. The occupational therapy group reported that the Generalised Anxiety Disorder Assessment (GAD-7) would be a sensible tool to measure this as it is already established in clinical practice. The GAD-7 is a valid and reliable 7-item instrument used to assess the severity of generalised anxiety disorder, commonly used in the NHS.^82 83^ As well as being valid and reliable, the GAD-7 is quick and easy to administer, which is an important facilitator.^66^ For these reasons, the GAD-7 would make an appropriate measure for the OBtAIN-PD. People with PD and care partners in this study stated that enjoying more meaningful activities was an important outcome for them. The occupational therapists report that improvement in clinical measures is an essential outcome for the OBtAIN-PD, and multiple participants suggested using the COPM. The COPM is a validated measure of a person’s self-perception of performance in everyday living and has been recommended for people with PD.^84^,^88^ The COPM has been used as a primary outcome measure in clinical trials of people with neurological conditions, including Parkinson’s.^47 89^ Furthermore, it has been shown to detect improvements with occupational therapy in people with mental health disorders, young people and older adults.^90^,^93^ This suggests that the COPM would be an appropriate outcome measure for use with OBtAIN-PD. In terms of service level outcomes, all participant groups indicated that preventing patients from being referred back to a community rehabilitation service within 6 months for the same issue is a critical outcome. Although a standardised measure for tracking this is not currently available, the data is routinely collected by services, making it feasible to monitor as part of a feasibility trial.

In summary, the OBtAIN-PD is a lifestyle management intervention delivered by occupational therapists. It will be delivered by occupational therapists in community-based rehabilitation teams and is underpinned by the ‘Do-Live-Well’ framework. The OBtAIN-PD will be measured using the COPM as the proposed primary outcome and GAD-7 as the proposed secondary outcome, which will allow the intervention to undergo initial testing (compared with usual occupational therapy care) as part of a proposed feasibility RCT. Given its multiple interacting components and the need for contextual tailoring, the OBtAIN-PD qualifies as a complex intervention.^34^

### Strengths and limitations of the study

The structured process to which participants contribute directly is a key strength of the coproduction approach, particularly when using logic modelling. The model illustrates how participants believe the intervention should be organised, including the necessary resources and expected outcomes. Online sessions provided a convenient method of participation, thereby facilitating engagement. We tailored our engagement during the sessions to suit the specific needs of the individual groups involved in this research project. This included offering group or individual sessions at times that were convenient for participants. This research may serve as a helpful foundation for future coproduction processes to be developed and evaluated. However, caution should be exercised when generalising specific aspects of our approach to other studies.

The sample size exceeded our target (n=34). Fewer care partners participated (n=9) than people with Parkinson’s (n=14) and occupational therapists (n=11), of whom we over-recruited. Though not substantial, the under-recruitment of care partners likely reflects the barriers they face participating in research, such as time constraints due to caring responsibilities.^94^ Future studies should aim to address these barriers more comprehensively, as providing a convenient participation method is not enough.^54 94^ The online sessions were convenient for participants and researchers, but the impact on recruitment cannot be overlooked; those with limited digital literacy may not have participated.^95^

Our sample was predominantly female, but this figure is skewed by the occupational therapy sample, which reflects the 90% female workforce.^96^ The sample was more evenly matched for people with PD. This was unexpected, especially as PD tends to be more prevalent in men, and men are over-represented in most research.^97^ Our findings may not fully represent the views of male people with PD, care partners or occupational therapists. It could be argued that this is less relevant for occupational therapists since the intervention design is centred on patient needs, rather than the clinician’s gender. Future studies should consider amending their recruitment strategy to address this imbalance. Despite our efforts, our sample across all groups mainly consisted of white English people from Christian or non-religious backgrounds. Other ethnic and religious denominations are represented in this study in small numbers, limiting external validity to other groups. Future studies should develop recruitment strategies to actively reach seldom-heard groups, as relying solely on peer referral methods like snowball sampling will likely produce limited outcomes.

## Conclusions

This study has systematically coproduced an intervention that occupational therapists can deliver to help people with PD live well with anxiety (OBtAIN-PD). The logic modelling approach provided a unique opportunity to coproduce the OBtAIN-PD with diverse participant stakeholders. These stakeholders included people living with PD (including care partners) and healthcare professionals. The identified desired inputs, processes and outputs for OBtAIN-PD resulted in a consolidated logic model. This provided the input for an intervention manual, information sheets and goal-planning documentation.

The logic model framework was a helpful tool for identifying the inputs needed for a successful intervention, the key processes essential for implementation and the outcomes that can be considered when determining the intervention’s impact. The OBtAIN-PD will undergo initial testing in a feasibility randomised controlled trial.

## Supplementary material

10.1136/bmjopen-2025-107930 online supplemental file 1

## Data Availability

Data are available on reasonable request.
