# Supplementary material for: Coproduction of an occupation-based complex intervention for living well with anxiety and Parkinson’s (OBtAIN-PD) using online logic modelling in the UK
Source: BMJ Open. 2026 Feb 22;16(2):e107930. doi: 10.1136/bmjopen-2025-107930 (PMC12927378; doi:10.1136/bmjopen-2025-107930)
Supplement: online supplemental file 1 [file bmjopen-16-2-s001.pdf]

Introduction

This manual will provide guidance for the provision of the **Occupation-Based Complex Intervention for living well with anxiety and Parkinson's Disease** (**OBtAIN-PD)** for the OBtAIN-PD feasibility cluster randomized controlled trial (RCT).

If your team has not been allocated to provide the OBtAIN-PD and you are providing usual care occupational therapy only, please stop reading this manual and inform the research team by emailing Jonathan.marsden@plymouth.ac.uk

This manual provides guidance and structure on how to deliver the OBtAIN-PD by an Occupational Therapist for a person with Parkinson's with anxiety but is not prescriptive as to the content of individual sessions.

Development of the OBtAIN-PD

The OBtAIN-PD project has its roots in clinical practice. The chief investigator (CI, Chris Lovegrove) was working in an inpatient ward for neurology with people with Parkinson's whose treatment was medically optimized, yet their participation in meaningful occupation remained substantially impeded. On assessment, these people were experiencing uncontrolled anxiety that was exacerbating their Parkinson's symptoms. A literature review revealed that there is currently no 'gold-standard' treatment for anxiety in Parkinson's and that current treatments seem to be less effective for people with Parkinson's. The CI became interested in exploring the development of a new intervention to help people with Parkinson's to live well with anxiety.

Formally spanning 2015 to 2023, the OBtAIN-PD project has consisted of four studies (excluding this feasibility trial) that have involved over 120 people with Parkinson's, care partners, and occupational therapists. These studies are:

- A foundational qualitative research study exploring the lived experience of people with Parkinson's (Lovegrove & Bannigan, 2021)
- A group concept mapping study to identify the key components of what would become the OBtAIN-PD (article in peer review)
- A scoping review to identify what occupational therapy interventions for adults with anxiety already exist, and what the characteristics of those interventions are (article in peer review)
- A study using an approach called 'logic modelling' to use the information from the above studies to co-produce the initial version of the OBtAIN-PD (article to be submitted for peer review in February/ March 2023)

Alongside these studies, we have conducted continuous Patient & Public Involvement (PPI) in the form of several consultations to involve people with Parkinson's, care partners, and occupational therapists in the development of our research studies. Using PPI can improve the quality and real-world relevance of research studies. We have conducted a range of PPI consultations to support this project, including a published article (Lovegrove *et al., 2017*) and an interactive session at the 2022 Royal College of Occupational Therapists' Annual Conference. This session was attended by over 100 occupational therapists.

The aim of this research project is to conduct a cluster randomized feasibility trial of the OBtAIN-PD in a real-world practice setting. We aim to use the data from this study to inform the design and planning of a future definitive trial, and to further develop and refine the OBtAIN-PD.

Overview of OBtAIN-PD sessions

The number of treatment sessions provided will depend on the person with Parkinson's goals.

The OBtAIN-PD trial protocol suggests eight one-hour sessions over a 10-week period based on published evidence. This is number of sessions **is not** prescriptive and is suggested for trial planning purposes. As the occupational therapist delivering the OBtAIN-PD you can decide how many sessions a patient receives (and over what time frame) to meet their goal based on your clinical decision-making,. For example, you may deliver all eight sessions (or more if you decide it is required) to meet the persons goal, or you may deliver only three sessions. There is no strict rule of how many sessions should be provided.

The below table provides an overview of activities performed the trial for the participants receiving the OBtAIN- PD based on the **suggested** eight one-hour sessions over 10 weeks as outlined in the trial protocol, and who will deliver the different activities. The different activities will be described in more detail in later sections.

Activities in the table are colour-coded according to who performs them. Green-shaded activities are performed by the community rehabilitation teams. Amber-shaded activities are performed by the CI (or delegated members of the research team).

Overview of activities (OBtAIN-PD)

Session/ Activity	Time	Activity (confirmed & suggested)	Delivered by?
Referral sent to the community rehabilitation team (CRT)			Normal referral routes- consultants, Parkinson's specialist nurses, clinics, other AHPs
Referral triaged as per local protocol	As per local protocol	• Referral triaged and prioritized<br>• First appointment booked<br>• Study details discussed	CRT clinician
Screening log completed, information pack sent to participant if they are interested	5 minutes maximum per potential participant	• Complete screening log<br>• If participant interested, send email of postal information pack (provided)	CRT clinician
Details sent to chief investigator	1-2 minutes	Participant details emailed to chief investigator<br><br>**Or**<br><br>CRT clinician sends details to CI at Christopher.lovegrove@nhs.net	Participant **or** CRT clinician
Screening and informed consent process completed	30 minutes	• Questions answered<br>• Informed consent documentation completed<br>• Participant details entered to trial database	Chief Investigator (Chris Lovegrove)
Baseline outcome measures taken	One week prior to first appointment<br><br>Clinical outcomes measures (60 minutes)<br><br>Patient-reported outcome measures (15 minutes)	• COPM<br>• eACS3<br>• GAD-7<br>• PDQ-39<br>• EQ-5D-5L<br>• Barthel Index<br>• Baseline clinical outcomes sent to the treating clinician	Chief Investigator (Chris Lovegrove)<br><br>Participant- Patient-reported outcome measures
Session 1	60 minutes (or as required by the patient), complete the	• Initial assessment and goal discussion	CRT occupational therapist

	therapist contact form (max. 5 minutes)	• complete the OBtAIN-PD goal planning sheet<br>• Provide 'Parkinson's & Anxiety' information sheet<br>• Complete Therapist contact sheet (see below)<br>• Report adverse events (if needed, see below)	
Session 2	60 minutes (or as required by the patient), complete the therapist contact form (max. 5 minutes)	• Review goal planning<br>• Treatment<br>• Provide necessary information sheet (exercise, sleep, or diet & nutrition) if indicated<br>• Amend goal sheet<br>• Complete Therapist contact sheet (see below)<br>• Report adverse events (if needed, see below)	CRT occupational therapist
Session 3	60 minutes (or as required by the patient), complete the therapist contact form (max. 5 minutes)	• Review goal planning<br>• Treatment<br>• Provide necessary information sheet (exercise, sleep, or diet & nutrition) if indicated<br>• Amend the goal sheet<br>• Complete Therapist contact sheet (see below)<br>• Report adverse events (if needed, see below)	CRT occupational therapist
Session 4	60 minutes (or as required by the patient), complete the therapist contact form (max. 5 minutes)	• Review goal planning<br>• Treatment<br>• Provide necessary information sheet (exercise, sleep, or diet & nutrition) if indicated<br>• Amend the goal sheet<br>• Complete Therapist contact sheet (see below)<br>• Report adverse events (if needed, see below)	CRT occupational therapist
Session 5	60 minutes (or as required by the patient), complete the therapist contact form (max. 5 minutes)	• Review goal planning<br>• Treatment<br>• Amend the goal sheet<br>• Complete Therapist contact sheet (see below)<br>• Report adverse events (if needed, see below)	CRT occupational therapist

Session 6	60 minutes (or as required by the patient), complete the therapist contact form (max. 5 minutes)	• Review goal planning<br>• Treatment<br>• Amend the goal sheet<br>• Complete Therapist contact sheet (see below)<br>• Report adverse events (if needed, see below)	CRT occupational therapist
Session 7	60 minutes (or as required by the patient), complete the therapist contact form (max. 5 minutes)	• Review goal planning<br>• Treatment<br>• Amend the goal sheet<br>• Complete Therapist contact sheet (see below)<br>• Report adverse events (if needed, see below)	CRT occupational therapist
Session 8	60 minutes (or as required by the patient), complete the therapist contact form (max. 5 minutes)	• Review goal planning<br>• Conclusion<br>• Complete Therapist contact sheet (see below)<br>• Report adverse events (if needed, see below)	CRT occupational therapist
12-week (from baseline assessments) follow-up	Clinical outcomes measures (60 minutes)<br><br>Patient-reported outcome measures (15 minutes)	As per baseline	Chief Investigator (Chris Lovegrove)<br><br>Participant- Patient-reported outcome measures
24-week (from baseline assessments) follow-up	Clinical outcomes measures (60 minutes)<br><br>Patient-reported outcome measures (15 minutes)	As per baseline	Chief Investigator (Chris Lovegrove)<br><br>Participant- Patient-reported outcome measures
Qualitative interview	60- 90 minutes	Semi-structured interview at a date and location convenient to the participant/ clinician	Chief Investigator (Chris Lovegrove)

A summary of both the clinical and patient-reported outcome measures is provided in *appendix 1*.

1. Initial assessment- delivered by CRT occupational therapist

Objectives

- Identify the person's occupational performance history, and how their current performance may differ from the past.
- Identify what values, roles, and interests give meaning to their activities.
- Identifying/clarifying the patient's perceived problems in activities and participation and priorities.
- Identifying/clarifying occupational therapy needs of care partner (if necessary).
- Examining and analysing aspects of the person, the activity, and the environment that hinder and promote meaningful participation. Identifying facilitating factors is important as they are used during treatment.
- Identify what factors influence the person's anxiety.
- Identify how anxiety affects the person's performance.
- Observe current occupational performance.
- Identify what the person wants to change and what they want to remain the same.
- Analysing the possibilities of changing the person, the activity, and the environment to positively influence participation.

Timing

The delivery of the initial assessment is flexible, depending on what suits the patient or carer best, and the occupational therapist's own style i.e., face-to-face, online, or telephone. Please follow the structure presented below when delivering the initial assessment. Some patients and care partners will want to rapidly do something and talk more about their issues along the way. Others will prefer to have an in-depth discussion first. As per usual practice, the initial interview will provide a complete picture of the referred issue to aid the occupational therapist in developing a treatment plan.

The chief investigator, Chris Lovegrove, will provide the outcomes of the baseline clinical outcome measures (COPM, eACS3) to the occupational therapist prior to the initial assessment appointment. During the initial assessment, the clinician will perform any additional clinical assessments that they deem necessary and check with the person that these are still the most important issues to address in sessions. This is important as goal setting is a core principle of the OBtAIN-PD approach.

As part of the OBtAIN-PD intervention, please complete the "OBtAIN-PD goal planning sheet". Ideally, this should be completed by the patient (if they are able) and retained by them. The purpose of this is to promote 'ownership' of the goals by the patient and to use this as a tool for future therapy sessions. Goals should be related to managing the impact of anxiety and engaging in meaningful activities. Paper copies of the goal planning sheet will be provided by the research team, as well as a link to an online version should more copies be required quickly. The goal planning sheet should be reviewed at the beginning and end of each session and adjusted accordingly, providing a new sheet if needed.

An example of a completed goal planning sheet with dummy data is included below.

OBtAIN-PD goal planning sheet
V.1 30.09.2022
IRAS ID: 318175

OBtAIN-PD

OBtAIN-PD goal planning

My long-term goal is:

To be able to be able to Walk my dog along the sea front without having a panic attack

I will accomplish this goal by the following date:

31st March 2023

This goal will be accomplished by taking the following steps:

(What short-term goals will you use to help you achieve your long-term goal? Example: "I will attend an exercise class at least two times a week")

- I will cut down the amount of coffee that I drink from four to two cups each day.
- I will stop drinking caffeine after midday.
- I will walk my dog to the end of my road with my wife.

The times that I feel I am at risk of not completing my goal and steps are:

(Situations that you know causes you have increased challenges with staying consistent. Example: "I have difficulty going to bed at a regular time because I feel pressured to go out")

- I tend to get a coffee whenever I go out with my family or friends because that is my 'go to' drink.
- I usually drink more coffee when I don't sleep well.
- When there are lots of other people out walking their dogs.
- When I don't know the route that we are going to walk.

Figure 1. Example of a completed goal planning sheet. This example is based on a real-life example given by a person with Parkinson's.

During the initial assessment and discussion on goals, your communications should focus on 'guiding' people in a compassionate manner to empower them to identify the steps to achieve their goals themselves. The purpose of this is to evoke motivation within the person to make positive change. To achieve this, when working with a person with Parkinson's the following communications skills should be used:

- ***Open-ended questions****:* using open-ended questions allows to find out more about the person's perspectives and ideas about how to change. These questions are also crucial in building and strengthening a collaborative therapeutic rapport. *Example: "How would you like things to be different?"*

- ***Affirming:*** this can be achieved through recognising and commenting on the person's strengths and abilities. Research in approaches such as motivational interviewing has shown that it helps rapport building by acknowledging the person's emotions and feelings. *Example: "This sounds like this has been really challenging. No wonder you have felt overwhelmed."*

- ***Reflective listening:*** Sometimes known as 'active listening', the purpose of reflective listening is to clarify what the person is saying for both the purposes of understanding correctly and to reflect to the person that they are being listened to. This can be useful in helping the person to consider change. *Example: "So I can check that I have understood what you have told me, you said…"*

- ***Summarising:*** this is closely linked to reflective listening. During summarising, the clinician reflects what the person has told them using their own words. This has the effect of the person 'hearing themselves' talk about change, which can be an effective motivator.

During the initial assessment, it is important to listen for 'change talk' to help guide the goal and treatment planning. Change talk are phrases that signal that the person is more willing, able, or ready to make a change. **The role of the clinician is to support the client to generate their change talk in a collaborative way, and to avoid imposing it.** Change talk can occur in several forms and can be exemplified by statements that indicate a desire for, ability to, reasons for, and a need to change. Some examples are included below:

- Desire statements indicate a desire to make a change e.g., *"Getting more exercise would make me feel so much better about myself."*

- Ability statements reflect the person's belief in their ability to make change e.g., *"With some help, I think I might be able to cut back on how much I eat."*

- Reason statements reflect the person's reasons for wanting to change e.g., *"I have to control my anxiety so I can spend time with my grandchildren."*

- Need statements indicate a need for change that might be more emotional than 'reason' statements (which tend to be more objective). E.g., *"My anxiety has to change, or my marriage will fall apart."*

During the session, reflecting this language back to the person when summarising can help to strengthen the person's commitment and motivation to change.

Some further examples of these communication skills are provided below.

	Examples
**Open-ended questions**	• "What makes you think it might be time for a change?"<br>• "What brought you here today?"<br>• "What happens when you [insert risky/problem/unhealthy behaviour]?"<br>• "What was that like for you?"<br>• "What's different about (quitting smoking, improving your exercise, diet, etc.) this time?"
**Affirming**	• "That's a good suggestion."<br>• "I appreciate that you are willing to meet with me today."<br>• "It sounds like it has been very hard to cut down on coffee."<br>• "It's clear that you're really aware of how important it is to get enough sleep."
**Reflective listening**	• "It sounds like…"<br>• "It seems as if…"<br>• "What I hear you saying…"<br>• "I get the sense that…"<br>• "I get the sense that this has been difficult…"<br>• "What I hear you saying is that your [insert risky/problem/unhealthy behaviour] is affecting you by…."
**Summarising**	• "It sounds like you are concerned that you are concerned about your anxiety because it is stopping you from spending quality time with your grandchildren."<br>• "You have been talking about improving your diet and losing weight as you feel it will help your anxiety. It seems you have started to recognise the less good things about being overweight. It's easy to understand why you want to work on this."

When completing the goal planning sheet, these communication skills should be used to support the person to complete the *“this goal will be accomplished by taking the following steps”* box. Examples of language to use to help the person decide on these steps include:

- “What changes were you thinking about making?”
- “How would you like things to turn out?”
- “After reviewing all of this, what’s the next step for you?”
- “It sounds like things can’t stay the same. What do you think you might do to change this?”
- “What are you going to do next?”
- “What do you think will help at this point?”

This is not an exhaustive list of phrases. You may have phrases that you routinely use in clinical practice. It is OK to use these as long as the conversation remains focused on guiding and eliciting from the person what they plan to do, rather than giving instruction or advising.

“What if the person wants a quick fix?”
It is common for people using health and care services to seek answers or fixes. OBtAIN-PD is intended to empower people with Parkinson’s to find their own solutions to living well with anxiety. In keeping with this, a simple phrase to help remind the person of their autonomy can be useful. An example would be; “You are the expert on yourself, so I’m not sure I am the best person to judge what will work for you. But I can give you an idea of what research evidence shows us and what other people have done in your situation.”

During the initial session, you should complete the standard paperwork that is required for your service. Following the session, you must complete a therapist contact sheet as described in section 3.

As part of the session, provide the person with the information sheet titled “Parkinson’s & Anxiety V1”. This sheet has been designed to provide introductory information about anxiety and Parkinson’s, something that people with Parkinson’s feel is important to receive at the beginning of the intervention to help them understand what is going on. Please talk through this sheet with the person, using some of the following questions to guide the conversation:

- Would you mind me telling you a little more about anxiety in Parkinson’s?
- (After reading the sheet) Are there any things raised in the information sheet that remind you of your anxiety?
- What things contribute to your anxiety?
- How does your anxiety change throughout the day?
- How does the environment affect your anxiety?
- How does your anxiety affect the things that you do?

You should document the session in the patient’s medical notes as per usual practice.

2. Treatment sessions

Purpose

To assist and support the patient and care partner in optimising meaningful participation by addressing barriers to engaging in meaningful occupation (as identified by the person with Parkinson's).

Treatment provision and timing

Treatment may take the form of education, advising and coaching, instructing, practising skills, and giving feedback. The potential content of treatment sessions is expanded on below.

The number of treatment sessions provided by the occupational therapist depends on the reason for referral, the patient's goals and the types of interventions delivered. This is a clinical decision made by the treating occupational therapist and is not prescribed.

The OBtAIN-PD approach is designed to be flexible in its application. Occupational therapy intervention should be tailored to each person; this is what creates dynamic and meaningful outcomes. Each person's treatment goal will be different, but the OBtAIN-PD provides a framework towards delivering this treatment, developed in co-production with people with Parkinson's, carer partners, and occupational therapists.

Information sheets

During the intervention, you may need to provide the person with extra information to help them adapt their roles, routines, and lifestyles to help them live well with anxiety. Three information sheets are available for you to give out; *"Exercise", "Sleep",* and *"Diet & nutrition".* These information sheets are based on currently published NHS guidelines. A summary of each information sheet is provided below.

Information sheet	Summary
Exercise	• Explains what exercise and physical activity is.<br>• Provides evidence-based recommendations for the amount per week (2.5 hours, 150 minutes).<br>• Gives examples of non-exercise activity
Sleep	• Explains what sleep is and why it is important.<br>• Provides NHS recommendations of how much sleep is needed per night (seven to nine hours).<br>• Provides suggestions on how to improve sleep for the person to refer to.
Diet & nutrition	• Provides general rules for following a healthy diet based on NHS recommendations.<br>• Provides information on medication, adapted cutlery, and swallowing problems to help inform the person's thinking.

The subjects and contents of these information sheets have been developed based on the thoughts and opinions of people with Parkinson's, care partners, and occupational therapists on what they felt should be included in the OBtAIN-PD. The information sheets **should not** be provided unless the topic has been identified by the person with Parkinson's, and **should not** be provided as a treatment. These information sheets are intended to aid the delivery of OBtAIN-PD by providing information and supporting discussion between the person and the occupational therapist.

Treatment
As part of the initial discussion, you will have supported the person to identify how they will accomplish their goal. Each treatment session should start by reviewing these steps and how the person has managed to work towards them.

Treatment should focus on directly addressing the person's own individual goal. For example, the goal used on the example goal planning sheet:

"To be able to walk my dog along the sea front without having a panic attack."

As opposed to 'talking' therapies such as cognitive behavioural therapy (CBT), OBtAIN-PD specifically focuses on engaging the person in their chosen meaningful occupation to remove barriers to participation. People with Parkinson's have expressed that they want their participation barriers addressed in pragmatic and direct ways.

Thus, this goal could be addressed in a variety of ways:

- Identifying the triggers that result in the person feeling anxiety and looking for ways to reduce them.
- Identifying parts of the activity that the person is already doing and encouraging them to complete that daily to increase their sense of mastery and confidence.
- Supporting the person to plan ahead to reduce potential stressors e.g., timing the walk when there are fewer people around.
- Graded exposure- supporting the person to engage in the activity at the location (in this case, a walk at the sea front), and gradually increasing this each session in collaboration with the individual.
- Situational use of relaxation strategies, such as breathing techniques, during the activity to prevent anxiety from escalating.
- Cueing strategies, such as auditory or visual cueing, to help with the management of freezing episodes or the initiation of movement.
- Cognitive support strategies, such as setting up reminder or prompts on mobile device, or planning novel (new and interesting) activities to promote cognitive initiation.

This is not an exhaustive list of the treatments provided by occupational therapists delivering the OBtAIN-PD. The list provides suggestions of the sorts of treatment activities that an occupational therapist may provide when working with a person with Parkinson's with anxiety.

You may use different treatments from the list above based on the individual's needs. Please provide usual occupational therapy intervention as needed e.g., providing compensatory equipment to reduce falls risk in ADLs. Usual occupational therapy intervention should form a maximum of 50% of

the session. For a description of usual care occupational therapy, please refer to the usual care manual (v1.1).

Session close and planning
The final part of each treatment session should focus on reviewing what has occurred during the treatment session, reviewing this in relation to the participant's goal, and planning the next session (if required). This process should include:

- Reviewing the person's experience of the session.
- Using the previously identified communication strategies to help the person identify what went well (and what didn't go so well).
- Plan the next session in collaboration with the person. For example, if the person walked their dog for 5 minutes, they may aim for 10 minutes in the next session.
- Updating the goal planning sheet if required.
- Confirm the date and time for the next treatment session.

Final session
If the person has completed their goal, and they do not require any further intervention, please review the goal planning sheet.

As part of the closing discussion, discuss with the person the steps that they have taken to accomplish their goal, and how they have addressed the potential barriers that they faced towards addressing their goal. Goal formation is a critical component in fostering behaviour change; closing the intervention by focusing on goal accomplishment can be a supportive factor in maintaining behaviour change.

To help maintain behaviour change, please discuss the following with the person:

- What are some habits that prevent you from doing the things that you want to?
- How do you react when you are not able to do the things that you want to?
- What helps you to develop new habits and routines?
- What are some barriers to making new habits?
- What are some unexpected things that may interfere with forming new habits and stop you from doing what you want to do? What steps can you take to prepare for such things?

Remember to tick the 'discharge' box on the therapist contact sheet (see below). This will indicate to the research team that this is the final treatment session.

Please discharge the person as per your team's process.

3. Therapist contact sheet and Adverse Event Monitoring

Monitoring how interventions are delivered is an important part of a clinical trial. This information is used to establish the average length of a treatment session, how many sessions are required on average, the most common treatments used, and any extra resources that might have been needed.

To help collect this information, the treating clinician will be required to complete a 'Treating Therapist Contact Sheet' (*appendix 2).*

This form has been designed to be completed remotely on a mobile work device in a matter of minutes. Once completed, the treating occupational therapist will be able to submit it directly to the trial database from the device. It is important that the contact sheet is completed promptly at the end of the session so that the information is as accurate as possible.

Paper copies will be provided so that the treating occupational therapist can log the session in the event of a device failure, or a mobile or internet provider fault. The information on the paper forms will need to be transferred to the online form as soon as is feasibly possible and submitted. Once submitted, the paper copy can be disposed of in a secure confidential waste bin.

Reportable adverse events (such as injurious falls, new panic attacks, or increases in freezing episodes) in a therapy session can be reported directly to the Peninsula Clinical Trials Unit (PenCTU) at the number provided on the form. This information will then be passed on to a co-investigator (Professor Jon Marsden) who will investigate in line with trial processes.

To further support the monitoring of adverse events, the participants will be required to complete a falls, adverse event, and social cost log every four weeks. This will be delivered electronically and completed online. A copy of this log can be seen in *appendix 3.*

Appendix 1: Outcome measures

Outcome measure	Use	Delivered by?
The Canadian Occupational Performance Measure (COPM)	The standard Canadian Occupational Performance Measure (COPM) is a valid measure of a person's self-perception of performance in everyday living. The COPM is a client-centred outcome measure for	Chief investigator (Chris Lovegrove) at baseline, 12-week,

	individuals to identify and prioritise everyday issues that restrict their participation in everyday living. The COPM is the proposed primary outcome for the future definitive trial	and 24-week follow-up.
Activity Card Sort (eACS3)	An assessment of a person's perceived level of participation with demonstrated applications in clinical practice and research.	Chief investigator (Chris Lovegrove) at baseline, 12-week, and 24-week follow-up.
Generalised Anxiety Disorder assessment scale (GAD-7)	A valid and reliable 7-item instrument used to assess the severity of generalised anxiety disorder. The GAD-7 is commonly used in the 'Improving Access to Psychological Therapies' pathway and will provide a viable way of comparing the OBtAIN-PD against other interventions in the future. A score of ≥10 identifies a level of anxiety that has a direct impact on the quality of life and is a recommended cut-off that identifies a need for further clinical evaluation.	Participant at baseline, 12-week, and 24-week follow-up.
European Quality of Life-5 dimensions (EQ-5D-5L)	Evaluation of health-related quality of life. This measure has been used in trials with people with Parkinson's and has been psychometrically validated for this population. The EQ-5D-5L can be used to calculate quality adjusted life-years (QALYs), enabling cost-utility analyses.	Participant at baseline, 12-week, and 24-week follow-up.
The Parkinson's Disease Questionnaire (PDQ-39)	This condition-specific questionnaire, a patient reported measure of health status and quality of life, has been psychometrically evaluated in people with Parkinson's.	Participant at baseline, 12-week, and 24-week follow-up.
Barthel Index	A self-reported scale used to measure a person's performance in activities of daily living and a widely used measure in research.	Participant at baseline, 12-week, and 24-week follow-up.
Fall, adverse event, and social cost log	A commonly used tool in research for measuring falls frequency and freezing of gait in people with Parkinson's, and will be used to measure adverse event (AE) rates in this study including AEs that are not falls such as panic attacks. Data regarding societal costs will be collected, such as unplanned loss of workdays for the participants and carers, and personal expenditure on support services.	Participants at baseline and then every four weeks after this.

Appendix 2 Treating Therapist Contact Sheet

Treating Therapists Contact Sheet (insert trial logo here)
V1 2.09.2022
IRAS ID: 318175
Sponsor: University of Plymouth
Chief Investigator: Chris Lovegrove

Participant study number: | | | | | |

Participant initials: | | | | | |

Date: D D / M M / Y Y

Time: H H : M M **AM** ☐ **PM** ☐

Session number: | | |

Type of contact: Home visit ☐ Online consultation ☐ Telephone consultation ☐

First visit ☐ Follow-up ☐ Discharge ☐

Location of contact: (Please enter location here)

Did you provide any equipment: Yes ☐ No ☐

What did you prescribe: (Please enter here)

What was covered in the session (brief summary or bullet points):

(Please enter here)

Total participant contact time: H H : M M

Total administration time: H H : M M

Any other comments: (Please enter here)

Safety Monitoring

If you become aware of any serious adverse event that has occurred because of the intervention delivery, please call the PenCTU Trial Managers on 01752 315256

[illegible]

(Insert trial logo)

Version 1.0 28.11.2022
IRAS Project ID: 318175

OBtAIN-PD DIARY

Monthly diary for week commencing: __ __ / __ __ / __ __ __ __

Region: LiveWell South West ☐ Royal Devon ☐

Participant ID: __ __ __ __

(Insert trial logo)

Version 1.0 28.11.2022
IRAS Project ID: 318175
Example Completed Diary

**Number of Falls**	None ☐	Less than usual ☐	About the same ☒	More than usual ☐	A lot more than usual ☐
**Number of falls that caused you an injury**	None ☒	Less than usual ☐	About the same ☐	More than usual ☐	A lot more than usual ☐
**Freezing of Gait episodes**	None ☐	Less than usual ☒	About the same ☐	More than usual ☐	A lot more than usual ☐
**Panic Attacks**	None ☐	Less than usual ☒	About the same ☐	More than usual ☐	A lot more than usual ☐
**Unplanned GP appointment**	None ☒	Less than usual ☐	About the same ☐	More than usual ☐	A lot more than usual ☐
**Unplanned hospital attendance**	None ☐	Less than usual ☐	About the same ☐	More than usual ☒	A lot more than usual ☐
**Unplanned social services visit**	None ☒	Less than usual ☐	About the same ☐	More than usual ☐	A lot more than usual ☐
**If you are in paid employment, have you had to reduce the number of hours you worked this week due**	No. I worked the same hours ☐		Yes. I have had to reduce my working hours ☐		Yes. I have had to stop work completely ☐

**to Parkinson's and anxiety?**			
**If you are not employed: due to your Parkinson's and anxiety, have you had to reduce the number of hours per week you spend carrying out your normal daily activities (e.g., gardening, housework, social activity)?**	No ☐	Yes. I have had to reduce my normal daily activities a little ☒	Yes. I have stopped my normal daily activities completely ☐
**Over the last week, has a relative or friend taken time off work to look after you?**	Not at all ☐	Yes, a little ☒	Yes. They had to stop work completely ☐
**Have you incurred any other costs because of your Parkinson's and anxiety this week?**	Yes ☐		No ☐
**If yes, please list them here.**	£20- raised toilet seat<br>£4- parking at hospital<br>Cancelled social event with friends		

4 weekly log

**Number of Falls**	None ☐	Less than usual ☐	About the same ☐	More than usual ☐	A lot more than usual ☐
**Number of falls that caused you an injury**	None ☐	Less than usual ☐	About the same ☐	More than usual ☐	A lot more than usual ☐
**Freezing of Gait**	None ☐	Less than usual ☐	About the same ☐	More than usual ☐	A lot more than usual ☐
**Panic Attacks**	None ☐	Less than usual ☐	About the same ☐	More than usual ☐	A lot more than usual ☐
**Unplanned GP appointment**	None ☐	Less than usual ☐	About the same ☐	More than usual ☐	A lot more than usual ☐
**Unplanned hospital attendance**	None ☐	Less than usual ☐	About the same ☐	More than usual ☐	A lot more than usual ☐
**Unplanned social services visit**	None ☐	Less than usual ☐	About the same ☐	More than usual ☐	A lot more than usual ☐
**If you are in paid employment, have you had to reduce the number of hours you**			Yes. I have had to reduce my working hours ☐		Yes. I have had to stop

(Insert trial logo)

Version 1.0 28.11.2022
IRAS Project ID: 318175

**worked this week due to Parkinson's and anxiety?**	No. I worked the same hours ☐		work completely ☐
**If you are not employed: due to your Parkinson's and anxiety, have you had to reduce the number of hours per week you spend carrying out your normal daily activities (e.g., gardening, housework, social activity)?**	No ☐	Yes. I have had to reduce my normal daily activities a little ☐	Yes. I have stopped my normal daily activities completely ☐
**Over the last week, has a relative or friend taken time off work to look after you?**	Not at all ☐	Yes, a little ☐	Yes. They had to stop work completely ☐
**Have you incurred any other costs because of your Parkinson's and anxiety this week?**	Yes ☐	No ☐	
**If yes, please list them here.**			
