# Supplementary material for: Coproduction of an occupation-based complex intervention for living well with anxiety and Parkinson’s (OBtAIN-PD) using online logic modelling in the UK
Source: BMJ Open. 2026 Feb 22;16(2):e107930. doi: 10.1136/bmjopen-2025-107930 (PMC12927378; doi:10.1136/bmjopen-2025-107930)
Supplement: Supplementary file 2 [file bmjopen-2025-107930.reviewer_comments.pdf]

PEER REVIEW HISTORY

BMJ Open publishes all reviews undertaken for accepted manuscripts. Reviewers are asked to complete a checklist review form and are provided with free text boxes to elaborate on their assessment. These free text comments are reproduced below.

ARTICLE DETAILS

Title (Provisional)

Co-production of an occupation-based complex intervention for living well with anxiety and Parkinson's (OBtAIN-PD) using online logic modelling in the UK

Authors

Lovegrove, Chris; Bannigan, Katrina; Marsden, Jonathan; Sturkenboom, Ingrid

---

VERSION 1 - REVIEW

**Reviewer**	**1**
**Name**	**Karlsson, Linn**
**Affiliation**	**Linkopings universitet**
**Date**	**05-Feb-2024**
**COI**	**No.**

Title

Developing an occupation-based complex intervention for living well with anxiety and Parkinson's (OBtAIN-PD) using logic modelling

Abstract

Please re-formulate objective and desig to clarify that this manuscript describe the procedure of designing an intervention for patients with PD and anxiety.

Introduction

Describe the diagnose criteria of PD.

(Line 54 – 55) Do 98,6% of the people with PD only have non-motor symtoms, or does they have non-motor symtoms in combination with motor symptoms? Please clarify.

(Line 55) Explain the abbreviation PWP.

(Line 78 – 79) Why is it important to consider the neurobiology which partly explain anxiety in PD, especially with regard to the intervention developed in this study?

Please include a summary of avaliable treatments and their effects for anxiety in combination with PD.

Materials and methods

Replace (XX) with the correct initals.

Please describe the logical modellig design more in detail, including a visual description of it. Please consider to describe the participants and recruitment of them before the design method. (Line 137) The information pack seem to lack summaries of systematical reviews (or RCTs) of treatment effects for PD and anxiety (in combination and respectively). If this is correct, please motivate why this not were included in the information to the participants.

Have you considered to illustrate the design process (stage 1 – 5) in a figure?

I lack information about education status in the demographics. Please include or motivate why it is not interesting if you do not have the information.

Results

I lack a presentation of the logic model (inputs – processes – outcomes) including the intervention components and outcomes (the intervention manual) that will be examined in the feasability RCT study that is mentioned in the manuscript.

Discussion

In general, the discussion is quite extensive. Consider to write it in a more condensed form. Please describe the logical modellig design elaborate about this with regard to generalizability.

The discussion include text about outcome measures. I miss this aspect previously in the text. Please consider to include something about outcome measuring in the result.

Elaborate about what in the intervention that is missing in treatments already available today: for example education, flexibility, relevant outcome measures, patient-centered focus, goals-setting et cetera. It seems to me that most of the things that the participant asked for already are included in treatment options for patients seeking care today. That is – what is new with this intervention?

In other complex conditions it is common with a multiprofessional approach. Please elaborate some about pros and cons with a multiprofessional intervention and why you choose to design a singe- professional intervention for such a complex condition as PD with anxiety.

VERSION 1 - REVIEW

**Reviewer**	**2**
**Name**	**Taghizadeh, Ghorban**
**Affiliation**	**Iran University of Medical Sciences, Department of Occupational Therapy**
**Date**	**10- Mar-2024**
**COI**	**No.**

We thank the authors for their comprehensive work on this article. This study skillfully deals with all aspects of the research, please write the parts of the study in a better order. The reader gets confused while browsing the article. We wish the authors continued success.

VERSION 1 - REVIEW

**Reviewer**	**3**
**Name**	**Kelly, Moira**
**Affiliation**	**Queen Mary University of London**
**Date**	**26-Jun-2024**
**COI**	**No.**

The management of anxiety in Parkinson's disease is an important topic in need of research. Occupational therapists have a potentially important part to play in the management of anxiety and research in this area is warranted. This paper describes the process of developing an intervention to reduce anxiety in people living with PD.

It would be useful to have some information on OT services for people with PD. Are OTs included in all clinical teams for people with PD? What proportion of people with PD get to see an OT? What is the role of OTs in the identification and management of anxiety in PD? Why are OTs best placed to undertake this intervention? Do OTs have training in anxiety management? This paper seems to refer to OT services in the UK. It would be useful to know more about services and clinical guidelines in other countries and any available evaluations.

The title refers to an 'occupation-focused intervention' which I initially took to mean work focused. 'Occupational therapy-focused' would be clearer.

The paper requires more detail on a number of areas.

I would suggest that more detail is required here on anxiety, with information on evidence for approaches used to manage anxiety such as behavioural approaches. OTs are in a good position to address anxiety pragmatically with people with PD in relation to lifestyle and day to day activities. It would also be useful to make some reference to research in other long-term conditions which address anxiety. For example, research in other LTCs have used psychological and behavioural interventions involving goal setting, patient education and self-management.

The description of the intervention requires more detail and support. What makes this

intervention complex with regard to the literature on complex interventions referred to by the authors? Whereas an 'end outcome' is given, the terms need to be defined e.g. 'meaningful activities'. Ideally primary and secondary outcomes of the intervention should be described.

Stage 1 – how was it decided who would be part of the project team? It would be good to have some detail on the different roles represented. Given that the management of PD is ideally multidisciplinary it would be helpful to identify the professional roles of the study team members. It would also be useful to identify the different areas of team expertise relevant to the development of a complex intervention. The heading for this section includes 'key objectives' but they are missing.

Stage 2 – what are the clinical activities that are being referred to? What did the information pack include?

What was involved in a session? (page 9) How long did they last? How were they structured? How many sessions did participants attend? Groups are referred to (p10). Did each participant attend more than one group session? How many participants had one-to-one sessions? What is a care partner? What does 'coherence' mean in this context? Why is it necessary to illustrate the interdependency of the individual components? Was the researcher facilitating the logic modelling process experienced in this area? It would be helpful to have more detail on the facilitation process. On page 11 it is stated that people with PD, care partners and OTs met separately in order to avoid bias. What are the implications of this for co-production (p13)? Did all the participants come together to consolidate the logic model? (p12).

What does fidelity testing mean here? What were the objectives of the testing and how was it carried out?

Sampling of participants with PD and OTs was through snowball sampling, but what was the process through which they were recruited? Information also needs to be given on how consent was gained.

Where was the research conducted?

What does the raw logic model mean? It is not clear how the raw statements were produced.

It seems odd to refer to resources as an input – all research and intervention will require resources. Is this specifically referring to OTs?

Figure 1 is not particularly helpful to the reader.

Figure 2 - The contents of the logic model require more explanation as to the 'chains of causes and effects' (p8). Activities such as, 'support, lifestyle management and information support' need to be clearly defined.

It's not very clear what the intervention to reduce anxiety will entail. Is it a behavioural intervention? What is the theoretical underpinning for the intervention? It is hard to see what will be tested in the proposed feasibility RCT. Is an educational intervention being developed? What will this entail? Is this for training OTs or people with PD? What does the intervention manual contain?

VERSION 1 - AUTHOR RESPONSE

Reviewer 1	
**Reviewer's comments**	**Author's response**
**Abstract** Please re-formulate objective and desig to clarify that this manuscript describe the procedure of designing an intervention for patients with PD and anxiety.	We have amended the objective and design as requested.
Describe the diagnose criteria of PD. (Line 54 – 55) Do 98,6% of the people with PD only have non-motor symtoms, or does they have non-motor symtoms in combination with motor symptoms? Please clarify. (Line 55) Explain the abbreviation PWP. (Line 78 – 79) Why is it important to consider the neurobiology which partly explain anxiety in PD, especially with regard to the intervention developed in this study? Please include a summary of avaliable treatments and their effects for anxiety in combination with PD.	We have added detail regarding the diagnostic criteria of Parkinson's. We have added clarity regarding people with PD living with motor and non-motor symptoms as requested. We have removed the abbreviation 'PWP' throughout the article and made sure this is changed to 'people with PD' where appropriate. We have added a brief summary regarding currently available treatments.
Replace (XX) with the correct initals.	We have replaced XX with all initials as requested.
Please describe the logical modellig design more in detail, including a visual description of it.	We have provided a visual description of a logic model in figure 2, titled "The blank logic model used with participants to co-produce the OBtAIN-PD." Please let us know if these are not displaying correctly in the final proof, and we will address this issue with BMJ Open. We have added additional detail as requested.

Please consider to describe the participants and recruitment of them before the design method.	We have moved the sections describing participant recruitment and our PPI activities to the beginning of the methods section and before the study design, as requested.
(Line 137) The information pack seem to lack summaries of systematical reviews (or RCTs) of treatment effects for PD and anxiety (in combination and respectively). If this is correct, please motivate why this not were included in the information to the participants.	We have added this detail as requested.
Have you considered to illustrate the design process (stage 1 – 5) in a figure?	We have included a figure of the study stages as requested. This is presented in figure 1.
I lack information about education status in the demographics. Please include or motivate why it is not interesting if you do not have the information.	We have added this detail as requested.
I lack a presentation of the logic model (inputs – processes – outcomes) including the intervention components and outcomes (the intervention manual) that will be examined in the feasability RCT study that is mentioned in the manuscript.<br><br>In general, the discussion is quite extensive. Consider to write it in a more condensed form.	We have presented the logic model in figure 3, titled "Consolidated logic model for the occupation-based complex intervention for living well with anxiety and Parkinson's (OBtAIN-PD)." We have included a copy of the OBtAIN-PD trial manual as supplementary material.<br><br>We have amended the discussion to make it more concise.
Please describe the logical modellig design elaborate about this with regard to generalizability.	We have added a statement discussing this as required.
The discussion include text about outcome measures. I miss this aspect previously in the text. Please consider to include something about outcome measuring in the result.	We have added additional detail to make this link clearer as requested.
Elaborate about what in the intervention that is missing in treatments already available today: for example education, flexibility, relevant outcome measures, patient-centered focus, goals-setting et cetera. It seems to me that most of the things that the participant asked for already are included in treatment options for patients seeking care today. That is – what is new with this intervention?	We have added a line clarifying this. The inclusion of the aforementioned concepts with a lifestyle management approach delivered by occupational therapists is the new concept with this intervention.

In other complex conditions it is common with a multiprofessional approach. Please elaborate some about pros and cons with a multiprofessional intervention and why you choose to design a singe-professional intervention for such a complex condition as PD with anxiety.	Thank you for highlighting this important part of the OBtAIN-PD. We have added discussion of these points.
**Reviewer 2**	
**Reviewer's comments**	**Author's response**
We thank the authors for their comprehensive work on this article. This study skillfully deals with all aspects of the research, please write the parts of the study in a better order. The reader gets confused while browsing the article. We wish the authors continued success.	Thank you for your feedback. We have amended the article throughout based on reviewer feedback whilst in keeping with the BMJ Open and structure as requested.
**Reviewer 3**	
**Reviewer's comments**	**Author's response**
It would be useful to have some information on OT services for people with PD. Are OTs included in all clinical teams for people with PD? What proportion of people with PD get to see an OT? What is the role of OTs in the identification and management of anxiety in PD? Why are OTs best placed to undertake this intervention? Do OTs have training in anxiety management? This paper seems to refer to OT services in the UK. It would be useful to know more about services and clinical guidelines in other countries and any available evaluations.	We have added additional detail as requested.
The title refers to an 'occupation-focused intervention' which I initially took to mean work focused. 'Occupational therapy-focused' would be clearer.	We have added detail to the introduction to clarify the terms 'occupation' and 'occupation-focused'. Whilst occupational therapists traditionally deliver occupation-focused interventions, they do not have to be delivered by occupational therapists. This is why we refer to an 'occupation-focused intervention.
I would suggest that more detail is required here on anxiety, with information on evidence for approaches used to manage anxiety such as behavioural approaches. OTs are in a good position to address anxiety pragmatically with people with PD in relation to lifestyle and day to day activities. It would also be useful to make	We have added some additional details regarding this as requested.

some reference to research in other long-term conditions which address anxiety. For example, research in other LTCs have used psychological and behavioural interventions involving goal setting, patient education and self-management.	
The description of the intervention requires more detail and support. What makes this intervention complex with regard to the literature on complex interventions referred to by the authors? Whereas an 'end outcome' is given, the terms need to be defined e.g. 'meaningful activities'. Ideally primary and secondary outcomes of the intervention should be described.	We have added detail at the end of the discussion as requested.
Stage 1 – how was it decided who would be part of the project team? It would be good to have some detail on the different roles represented. Given that the management of PD is ideally multidisciplinary it would be helpful to identify the professional roles of the study team members. It would also be useful to identify the different areas of team expertise relevant to the development of a complex intervention. The heading for this section includes 'key objectives' but they are missing.	We have added detail to this section, including the key objective in the final line.
Stage 2 – what are the clinical activities that are being referred to? What did the information pack include?	Clarification regarding clinical activities has been added. Details regarding the information pack contents have also been included.
What was involved in a session? (page 9) How long did they last? How were they structured? How many sessions did participants attend? Groups are referred to (p10). Did each participant attend more than one group session? How many participants had one-to-one sessions? What is a care partner? What does 'coherence' mean in this context? Why is it necessary to illustrate the interdependency of the individual components? Was the researcher facilitating the logic modelling process experienced in this area? It would be helpful to have more detail on the facilitation process. On page 11 it is stated that people with PD, care partners and OTs met separately in order to avoid bias. What are the implications of this for	The structure, average length of, and number of sessions has been added.<br><br>The term 'coherence' has been clarified.<br><br>The number of participants who had a one-to-one session has been added as requested to aid transparency.<br><br>The term 'care partner' has also been clarified.<br><br>Details regarding facilitator training have been added.

co-production (p13)? Did all the participants come together to consolidate the logic model? (p12).	The term 'interdependency' has been replaced with 'interaction' as we feel this is clearer. We have added detail to explain that this is linked to the intended outputs.<br><br>Detail has been added to explain that participants from all groups contributed to the consolidation of the final logic model.
What does fidelity testing mean here? What were the objectives of the testing and how was it carried out?	Thank you for highlighting this. We have added detail to explain that the fidelity testing was a future event and not part of this trial. We have also added detail to explain the term.
Sampling of participants with PD and OTs was through snowball sampling, but what was the process through which they were recruited? Information also needs to be given on how consent was gained.	We have added detail regarding sampling and recruitment. Consent information is included in the 'Ethical Considerations' section.
Where was the research conducted?	We have added this detail to the 'Design' section.
What does the raw logic model mean? It is not clear how the raw statements were produced.	We have removed the 'word' raw to avoid confusion. We have also added clarity that these statements were generated based on the participants ideas.
It seems odd to refer to resources as an input – all research and intervention will require resources. Is this specifically referring to OTs?	We have added detail to this section.
Figure 1 is not particularly helpful to the reader.	We have removed figure 1 as suggested.
Figure 2 - The contents of the logic model require more explanation as to the 'chains of causes and effects' (p8). Activities such as, 'support, lifestyle management and information support' need to be clearly defined.	Figure 2 has been retitled as Figure 1.<br><br>We have added details regarding the causal chain.<br><br>Details have been added regarding 1:1 support, lifestyle management, and information support/provision.
It's not very clear what the intervention to reduce anxiety will entail. Is it a behavioural intervention? What is the theoretical underpinning for the intervention? It is hard to see what will be tested in the proposed feasibility RCT. Is an educational intervention being	We have added detail regarding the theoretical underpinning of the OBtAIN-PD. Additionally, we have added a short section at the end of the discussion summarising the OBtAIN-PD.

developed? What will this entail? Is this for training OTs or people with PD? What does the intervention manual contain?	Details about the contents of the intervention manual have been included pages.
